# Association of Changes in Primary Care Spending and Use With Participation in the US Affordable Care Act Health Insurance Marketplaces

**DOI:** 10.1001/jamanetworkopen.2020.7442

**Published:** 2020-06-10

**Authors:** Sungchul Park, Jim P. Stimpson, Giang T. Nguyen

**Affiliations:** 1 Department of Health Management and Policy, Dornsife School of Public Health, Drexel University, Philadelphia, Pennsylvania; 2 Harvard University Health Services, Harvard University, Boston, Massachusetts

## Abstract

This cross-sectional study examined whether the US Affordable Care Act (ACA) Marketplace was associated with changes in primary care spending and use.

## Introduction

The US Affordable Care Act (ACA) Health Insurance Marketplaces have been associated with improvements in access to care,^1^ but evidence on primary care is limited.^2^ There is evidence that suggests restricted hospital networks as well as low primary care physician participation in the Marketplaces^3,4^ may limit access to primary care services. Therefore, we examined whether the ACA Marketplace was associated with changes in primary care spending and use.

## Methods

We used nationally representative data from the 2010-2017 Medical Expenditure Panel Survey. We included adults 26 to 64 years of age with family incomes between 138% and 400% of the federal poverty level in our sample. The intervention group was defined as persons with private nongroup insurance coverage who were eligible for Marketplace coverage with premium subsidies and/or cost-sharing reductions. Persons with employer-sponsored insurance were defined as the control group because a negligible association between the ACA and employer-sponsored insurance was found.^5^ Outcome measures included annual primary care spending, out-of-pocket spending for primary care visits, any primary care visit, and number of primary care visits. Spending was inflation-adjusted to 2017 US dollars. We defined the pre-ACA period as being from 2010 to 2013 and the post-ACA period as being from 2014 to 2017. Control variables included age, sex, race/ethnicity, marital status, educational level, family income, metropolitan residence, self-reported health status, number of chronic conditions, and survey year.

We used difference-in-difference multivariate linear regression (annual primary care spending, out of pocket spending for primary care, and number of primary care visits) and linear probability (any primary care visit) models that were adjusted for survey weights and robust standard errors. We conducted sensitivity analysis by limiting the intervention group in the post-ACA period to those who gained Marketplace coverage. We also conducted a sensitivity analysis by using a 2-part model for annual primary care spending and out-of-pocket spending for primary care visits, a negative binomial model for primary care visits, and a logistic model for any primary care visit. Because the distribution of health care spending is skewed, we also conducted a sensitivity analysis by reexamining the linear regression model with log-transformed spending. This study used deidentified, publicly available data and therefore was considered nonhuman subjects research. The study followed the Strengthening the Reporting of Observational Studies in Epidemiology (STROBE) reporting guideline. All analyses were conducted using Stata statistical software version 16.0 (StataCorp). All *P* values were from 2-sided tests, and the results were deemed statistically significant at *P* < .05.

## Results

We included 39 415 adults (19 274 women [48.9%]; mean [SD] age, 42.4 [11.8] years). Our analysis showed no significant changes in annual primary care spending, out-of-pocket spending for primary care visits, or any primary care visits between the intervention and control groups (Table 1). We found a statistically significant, but modest, increase in the number of primary care visits among the intervention group relative to the control group (differences-in-differences adjusted estimate, 0.24 [95% CI, 0.11-0.38]; *P* < .001). Our findings were robust to sensitivity analyses (Table 2). The parallel trends assumption for the difference-in-difference analysis was met for all outcomes.

**Table 1.** Difference-in-Difference Model for Primary Care Spending and Use Among Adults Eligible for the ACA Marketplace Relative to Adults With Employer-Sponsored Insurance

Outcome	Mean (SD) value				Differences-in-differences adjusted estimate^a^	
	Intervention (N = 3563)		Control (N = 35 852)			
	Pre-ACA period (2010-2013)	Post-ACA period (2014-2017)	Pre-ACA period (2010-2013)	Post-ACA period (2014-2017)	Estimate (95% CI)	*P* value
Primary care spending, $	225.05 (1099.67)	263.88 (783.20)	270.38 (978.73)	227.86 (1485.10)	44.96 (−24.21 to 114.14)	.20
OOP spending for primary care visits, $	50.35 (128.15)	45.60 (117.89)	40.31 (121.52)	34.13 (126.89)	−2.89 (−13.72 to 7.93)	.60
Any primary care visit, %	0.49 (0.50)	0.50 (0.50)	0.51 (0.49)	0.45 (0.49)	0.02 (−0.01 to 0.06)	.24
No. of primary care visits	0.95 (1.44)	1.20 (1.89)	1.15 (1.90)	0.99 (1.66)	0.24 (0.11 to 0.38)	<.001

Abbreviations: ACA, Affordable Care Act; OOP, out-of-pocket.

^a^ Differences-in-differences adjusted estimate was estimated from a linear regression model or a linear probability model that controlled for age, sex, marital status, educational level, family income, area of residence, self-reported health status, 10 chronic conditions (asthma, arthritis, diabetes, emphysema, stroke, heart attack, coronary heart disease, other heart disease, angina, and joint pain), and survey year.

**Table 2.** Sensitivity Analyses for Difference-in-Difference Model for Primary Care Spending and Use Among Adults Eligible for the ACA Marketplace Relative to Adults With Employer-Sponsored Insurance

Outcome	Differences-in-differences adjusted estimate	
	Estimate (95% CI)	*P* value
Limiting to individuals who gained Marketplace coverage^a^		
Primary care spending, $	43.76 (−28.26 to 115.78)	.23
OOP spending for primary care visits, $	−8.66 (−25.10 to 7.77)	.30
Any primary care visit, %	−0.01 (−0.07 to 0.06)	.87
No. of primary care visits	0.19 (0.01 to 0.38)	.03
Using different models^b^		
Primary care spending, $	47.76 (−59.42 to 154.95)	.49
OOP spending for primary care visits, $	−2.44 (−29.77 to 24.86)	.61
Any primary care visit, %	0.05 (−0.01 to 0.11)	.53
No. of primary care visits	0.27 (0.08 to 0.46)	<.001
Using linear regression model with log-transformed spending^a^		
Primary care spending, log ($)	0.50 (−0.10 to 1.10)	.10
OOP spending for primary care visits, log ($)	−0.05 (−0.59 to 0.47)	.82

Abbreviations: ACA, Affordable Care Act; OOP, out-of-pocket.

^a^ Differences-in-differences adjusted estimate was estimated from a linear regression model or a linear probability model that controlled for age, sex, marital status, educational level, family income, area of residence, self-reported health status, 10 chronic conditions (asthma, arthritis, diabetes, emphysema, stroke, heart attack, coronary heart disease, other heart disease, angina, and joint pain), and survey year.

^b ^ Differences-in-differences adjusted estimate was estimated from a 2-part model, a logistic model, or a negative binomial model that controlled for age, sex, marital status, educational level, family income, area of residence, self-reported health status, 10 chronic conditions (asthma, arthritis, diabetes, emphysema, stroke, heart attack, coronary heart disease, other heart disease, angina, and joint pain), and survey year. Using the marginal effects, the mean values of the outcomes for each group in the pre-ACA and post-ACA period were estimated, and then postestimation tests were conducted to estimate the differences in the outcomes between adults eligible for the ACA Marketplace and adults with employer-sponsored insurance before and after the ACA.

## Discussion

There were no or little changes in primary care spending and use after the implementation of the ACA Health Insurance Marketplaces in the US. The null findings might be explained by the poor incentives for primary care associated with the low dollar value of cost-sharing subsidies; newly enrolled individuals, especially those with low health literacy,^6^ might be unaware of the new system; or low participation by primary care physicians’ in Marketplace plans may not be increasing perceived access to care.^4^ Our findings suggest that the provisions in the ACA to improve access to primary care may not be sufficient. The interpretation of this study should be viewed within the limitations of the data, which includes that our study used cross-sectional data and that there may be no perfect control group for the ACA Marketplace.^5^
